# A sociotechnical approach to smartphone research: outline for a holistic, qualitative mobile method

**DOI:** 10.1177/1329878X241253011

**Published:** 2024-05-15

**Authors:** Stephanie Ketterer Hobbis, Geoffrey Hobbis

**Affiliations:** Sociology of Development and Change, 593528 Wageningen University, Wageningen, the Netherlands; Knowledge, Technology and Innovation, 593528 Wageningen University, Wageningen, the Netherlands

**Keywords:** smartphones, ethnography, body techniques, anthropology of technology, digital diversity, mobile methods, Solomon Islands

## Abstract

Smartphones have become crucial for understanding how digital technologies are adopted and adapted into people's lives, while also emerging as tools for studying social phenomena more broadly. Drawing on insights from our own longitudinal work in Solomon Islands, this article details a sociotechnical approach to smartphone research that combines both potentialities. It distinguishes itself from other smartphone-based methods by connecting media-centric perspectives with non-media-centric approaches through an additional focus on body techniques. The approach is centered on object-centric, semi-structured interviews embedded in longitudinal participant observation and theoretically informed by anthropologies of technologies. Emphasizing a holistic perspective and the diversity of human experiences, this approach allows for generating material evidence of contextually-embedded mediations of social relationships through the hardware and software of the phones themselves.

## Introduction

A pocket-sized complex computer or ‘metamedium’ (Humphreys et al., 2018), the smartphone ‘[affects] many dimensions of human existence: the ontological as well as the anthropological’ (Fortunati, 2023: 19), what we do and how we understand and relate to it. Even in the most infrastructurally disconnected areas like the big ocean states of the southwestern Pacific, where most of our empirical research has focused on, they are entangled with core aspects of everyday life. They have shifted, among many other things, how languages are being used (Vandeputte-Tavo, 2013), how children are being raised (Horst, 2021), and more broadly, the moral frameworks in which social, political and economic relationships are formed, ruptured, strengthened and maintained (Hobbis, 2020; Horst and Foster, 2023).

In this context of global ubiquity alongside global diversity, scholars across the social sciences and humanities have been grappling with how to engage with the smartphone. They have sought to understand it both as a subject of inquiry in its own right *and* as a tool for data collection, for studying social phenomena far and wide.^1^ They have recognized that smartphones may, technologically, be comparable irrespective of where and by whom they are used,^2^ but also that a baseline technological comparability in design does not necessitate comparability in usage. On the contrary, smartphone researchers have demonstrated how people around the world, from urban Tokyo (Ito et al., 2006) to rural Papua New Guinea (Watson and Duffield, 2016), have made smartphones their own, fitting their use into context-specific needs, interests and values.

This ubiquity alongside diversity is made even more complicated by the fact that, in any given context, ‘there are so many pieces of everyday life and dimensions of… existence that are encompassed by the smartphone that it is difficult to list them’ (Fortunati, 2023: 20) and that it is even more difficult to examine them comprehensively without careful methodological consideration. Because of their ubiquity and diverse implications for everyday lives (nearly) everywhere, smartphones necessitate ‘methodological innovation to ensure empirical validity in a changing world’ (Kaufmann, 2018: 234). Researchers have to find ways to engage, or at least acknowledge, the entanglements of smartphones with everyday life and to do so in ways that acknowledge that smartphones are not only sources of data, but also tools for data collection, in and of themselves (cf Boase and Ling, 2013; Boase and Humphreys, 2018).

Based on a decade of dedicated smartphone-centric and context-specific research in Solomon Islands, this article describes a qualitative, sociotechnical approach to understanding smartphones. This approach is designed to both explicitly tease out diverse experiences and encounters with the smartphone and to account for its methodological potentials and pitfalls. It does so by furthering efforts in digital research towards ‘holistic contextualisation’ (Miller, 2019: 1097). Additionally, it teases out diversity by combining insights from media-centric research on particular technologies, platforms or apps (cf Montfort and Bogost, 2020), including the smartphone itself (cf Fortunati, 2023), with non-media centric approaches that focus on the everyday embedding of particular, or a collection of, digital technologies (cf Miller et al., 2016, 2021). Leaning primarily on French contributions to the anthropology of technology (cf Coupaye, 2013; Lemonnier, 2012, 2018) while also drawing on broader, international debates in the field (cf Brunn and Wahlberg, 2022; Pfaffenberger, 1992), the approach we propose here is specifically designed to do both. As we detail in the following, our sociotechnical approach is media-centric in its technological focus on smartphones and non-media-centric in its emphasis on everyday entanglements including recognition of other technologies. Simultaneously, we connect the two perspectives with an additional focus on body techniques.

## Empirical foundations: perspectives from Solomon Islands

We have developed and tested this approach for and during longitudinal research in Solomon Islands. In Solomon Islands, mobile phones started proliferating in 2010 when a telecommunications monopoly fell at approximately the same time as hardware costs were significantly reduced (cf Hobbis, 2019a). Reflecting the appeal and reach of smartphones, Solomon Islanders have since adopted and adapted the technology widely and quickly. This is the case even though the vast majority of the population only has irregular access to the cash income necessary to purchase mobile phones, and even less so, to connect them to a telecommunication network (cf Hobbis, 2020).

Since 2014 we each conducted a total of 15 months of ethnographic fieldwork in the country with a focus on rural and urban Lau-speakers of North Malaita (cf Hobbis, 2017, 2020; Hobbis and Hobbis, 2022a) but also including members from other language groups (cf Inone et al., 2021). We have also been continuously engaging with Solomon Islands, Lau-speakers and other Solomon Islanders through social media, especially Facebook, as the primary digital platform for Solomon Islands Internet users. Simultaneously, our work in Solomon Islands has addressed both dimensions of smartphone research. We have studied diverse adaptations of smartphones, specifically, among the Lau (cf Hobbis, 2020) and we have researched broader transformations in Lau lives that are, given the technology's ubiquity, increasingly entangled with its use (cf Hobbis et al., 2024).

Solomon Islands is well suited to generate new insights into the globally diverse smartphone, in particular because it does not easily compare to the often ‘developed’ but also urban contexts that dominate smartphone research (cf Hobbis et al., 2023). Over 60 languages are spoken in this archipelago state with a population of about 700.000, spread across six main islands and many hundreds smaller ones. Solomon Islanders largely live in rural areas—still around 80%—and often have locally varied, limited and unreliable access to state infrastructures such as roads (Hobbis, 2019b) or also telecommunication towers and the services they are meant to provide (Hobbis, 2018).

Highlighting how insights from Solomon Islands can disrupt dominant discourses in media research, we found that Greenfield's claim that *all* smartphones are ‘useless unless activated by a commercial service provider’ (2018: 17) by no means holds true. On the contrary, the Lau regularly uses smartphones to consume and produce multimedia files without ever, or rarely, being connected to the world wide web (Hobbis, 2020; Hobbis and Hobbis, 2022b), showcasing possibilities for smartphone use far beyond those of commercial connectivity. Our research has also challenged dominant debates in digital economic research (e.g., Srnicek, 2017), which postulates that especially platforms such as Facebook, as they are used on smartphones, bring industrial-capitalist systems and values even to the most remote areas, such as rural Solomon Islands. Drawing on our sociotechnical approach we were able to refute this claim, demonstrating instead the emergence of a ‘platform horticulturalism’ (Hobbis and Hobbis, 2022a) and of digital bush markets that are centered on reciprocal economic values (Hobbis and Hobbis, 2023).

Doing smartphone research in a place like Solomon Islands also quickly highlights pragmatic and ethical problems with existing methodological approaches to smartphone research (cf Beaulieu, 2022). In a postcolonial context with a long, and ongoing history of (attempted) extractive exploitation by foreign actors (cf Allen, 2017), Solomon Islanders are not readily willing to participate, for instance, in passive data collection via a smartphone app. On the contrary, as we have argued elsewhere (Hobbis and Hobbis, 2017, 2022a), Solomon Islanders variously resist automated digital data collection, be it on Facebook or through biometric registration technologies. Instead, they participate in digital research mostly only based on individual relationships of trust with researchers themselves. In other words, irrespective of the empirical effectiveness of passive data collection (cf Boase and Ling, 2013), in places like Solomon Islands and, more broadly, among populations reasonably worried about data misuse (cf Keusch et al., 2021), ethical, and empirically sound, smartphone research, even when partially done via social media (cf Horst and Foster, 2023; Hobbis et al., 2024), requires deep contextual and relational embedding.

Valuing this need for contextual, relational embedding our research does not claim to be representative of Solomon Islands and/or Solomon Islanders at large. Instead, it provides snapshots of the digital lives of the people we encountered, as they are adopting and adapting smartphones in the broader context of Solomon Islands and its global entanglements. We showcase their experiences, and how they relate to broader global debates in media research. Designed for, and perpetually refined based on empirical work in Solomon Islands, our sociotechnical approach, thus, sets out to strengthen smartphone research in *diverse* environments. Our goal with this article is to advance this approach in media studies at large as part of broader, often ethnographic engagements with globally diverse digital, smartphone-led transformations (cf Foster, 2024; Horst and Foster, 2023; Miller, 2018; Tenhunen, 2018). By providing a ‘step-by-step’ outline of our sociotechnical approach combined with snippets of our relevant empirical work, we seek to provide a methodological, adaptable steppingstone to other smartphone researchers in diverse contexts. Simultaneously, by providing this in-depth discussion of our sociotechnical approach, we aim to increase the methodological transparency of smartphone research that relies on qualitative means of data collection, participant observation and interviews in particular, encouraging other qualitative researchers to do the same.

## A sociotechnical perspective

Media-centric in its technological focus on smartphones and non-media-centric in its emphasis on holistic contextualization, our sociotechnical approach to smartphone research builds on insights from digital material culture studies (cf Miller, 2018; Miller et al., 2016) and non-media-centric media studies (cf Krajina et al., 2014; Morley, 2009). These two approaches recognize smartphones and digital technologies more broadly as ‘cultural products that exist in the social and political worlds within which they were developed [are distributed and used]’ (Wilson and Peterson, 2002: 462). They argue that digital technologies ‘are not exempt from the rules and norms of those worlds’ (Wilson and Peterson, 2002: 462; cf Tenhunen, 2018) as they transform human existence ontologically and anthropologically.

The two approaches seek to understand contextual differences—and similarities—in ‘who has access to what, how that access is patterned and what consequences that access has for everyday experiences’ (Krajina et al., 2014: 668); and they do so by emphasizing the materiality of smartphones, arguing against artificial separations between the ‘virtual’ and the ‘real’ in the smartphone age. They counter existing biases about the impact of smartphones and open the door to a non-deterministic, materially-grounded study of digital worlds, for example, by revealing moments of both involuntary and purposeful disconnection from digital technologies and infrastructures (cf Pype, 2021).

Our sociotechnical approach shares a similar emphasis on subject-object relations and their everyday embedding. However, we also propose an extension to these dominant perspectives, inspired by French contributions to the anthropology of technology.^3^ French proponents of the anthropology of technology have argued that research on technologies at large (not just digital ones) has too often failed to effectively engage with the ‘body techniques’ (Mauss, 2006) that are central to shaping subject-object relations and, as a result, context-specific lifeworlds more broadly. ‘Body techniques’ or the ‘material actions performed by human beings’ (Lemonnier, 2018: 171) are here defined as an ‘[extension of] human bodily and cognitive capacities in order to perform tasks or a set of tasks, or more generally to engage with the world’ (Coupaye, 2021: 49). Body techniques are ‘always themselves social phenomena and are always systematic’ (Brunn and Wahlberg, 2022: 5; cf Lemonnier, 2012). They require in-depth investigation to understand not only particular technologies but also ‘basic human logics, social relationships, and societies’ (Brunn and Wahlberg, 2022: 13; cf Lemonnier, 2012). In other words, French approaches to the anthropology of technology argue that to understand the role of technologies in everyday life, it is essential to engage with the body techniques that reveal broader ‘material and social processes that form, for example, through gardening… or smartphones’ (Brunn and Wahlberg, 2022: 5).

As essentially *mobile* technologies, established qualitative approaches to smartphone research have recognized the embodied dimensions of smartphones. They have theorized smartphones as ‘*relational to* and *part of* a world in movement’ (Pink and Fors, 2017: 221; emphasis in original) and, thus, as ‘catalyzing new forms of corporeality, new embodiments, new ways of knowing and being human’ (Ellsworth, 2005: 126). However, rarely has this recognition of embodiment and body techniques more specifically been integrated into smartphone research as equivalent to, and firmly entangled with, material culture and everyday life – or at least it has not been acknowledged as such in relevant methodological reflections. As is the case with much other technological research, body techniques have too often disappeared in the background of the ‘obviousness of material culture and the triviality of everyday life’ (Coupaye, 2013: 74); or, shifting the balance the other way, body techniques are foregrounded to such an extent that non-media centric dynamics are overshadowed by a focus on particular technological, bodily features such as self-tracking (cf Pink and Fors, 2017).

In comparison, the sociotechnical approach to smartphone research that we detail in the following considers the essential entanglements of material cultures, body techniques and sociotechnical systems following the non-digital insights of French anthropologists of technology. Ludovic Coupaye (2013), for instance, demonstrated how the lifeworlds of the Abelam of Papua New Guinea are condensed into yams through the various material cultures, techniques and sociotechnical systems that encompass their production, distribution and consumption. Among the Abelam, yams are, in essence, compositional objects. They explicate the sociocultural construction of technological processes. Besides, material actions surrounding yams situate human actors morally. They allow for determining morally acceptable, and morally transgressive, behaviours and mediate not only relations between the living but also between the living and the dead. In other words, they reveal fundamental insights into Abelam social relations and values.

As Geoffrey has argued elsewhere (Hobbis, 2020), when approached from such a sociotechnical perspectives, smartphones are analytically even more intriguing than compositional objects such as Abelam yams. Compositional objects condense social networks and cultural meanings on a symbolic but not a technological and material level. The sociotechnical embedding of yams among the Abelam does not easily compare, for instance, to the role of yams in Denmark, where rye bread assumes moral significance instead (cf Karrebaek, 2014). Smartphones, on the other hand, appear comparable cross-culturally. They bridge social networks and cultural meanings on a sociocultural *and* technological level. They are *supercompositional* objects (Hobbis, 2020).

By their very design, smartphones use the force of digital computation to materially condense tracings of social networks and their moral dimensions including (un)certainties into SIM cards, as exemplified in widespread debates about morally good social relations (cf Huang, 2018; Hobbis and Hobbis, 2022b). Smartphones further condense cultural meaning, for example, in the form of multimedia stored on MicroSD cards, which materialize and reconstitute these relations digitally (cf Crowdy and Horst, 2022). They do so, among others, in photographs that ‘cannot be contained in the relation between the visual and its material support but rather through an expanded sensory realm of the social in which photographs are put to work’ (Edwards, 2012: 228). In other words, through the sociotechnical systems in which they are embedded, smartphones condense aspects of the society and culture in which they are used regardless of the historical particularities of the people who produce, distribute and use them. Yet, at the very same time, due to contextually unique material cultures, techniques and sociotechnical systems, they also reflect the values and anxieties of these people.

Accordingly, smartphones are particularly well suited as subjects of sociotechnical analysis, allowing not only for an examination of their significance in everyday life, but also of everyday life by means of understanding the material actions that surround smartphones in a given context. We have shown elsewhere how this, for example, can lead to a better understanding of diverse economic systems in the smartphone age (cf Hobbis and Hobbis, 2022a, 2023). In this article we focus less on these conceptual contributions to smartphone research. Instead, we describe, based on snippets from our own work, how to operationalizes this approach to facilitate better understandings of diversity in the smartphone age, and to broader discussions about the roles of smartphones as a (qualitative) research tool.

## Towards a holistic, qualitative mobile method

Reflecting broader ethnographic traditions, two methods are at the core of our sociotechnical approach to smartphone research: participant observation and semi-structured interviews. To realize the sociotechnical promise of both methods, our approach focuses specifically on uncovering context-specific body techniques, material cultures and sociotechnical systems as they pertain to smartphones but also life more broadly. They combine non-media centric and media-centric perspectives in one (more) holistic, qualitative mobile method. This method may equally be deployed to interrogate the role of smartphones in everyday life, or to understand other aspects of everyday life as it now also includes the smartphone in diverse settings.

### Participant observation

Participant observation, or co-located, co-presence wherein the researcher, in as much as possible participates in, and observes, body techniques of everyday life, is the cornerstone for uncovering particular sociotechnical systems. These everyday embodied entanglements reveal ‘*heterogeneous* constructs that stem from the successful modification of social and non-social actors so that they work together *harmoniously*—that is, so that they resist dissociation’ (Pfaffenberger, 1992: 498; emphasis added). In other words, by variously participating in, and observing, very context-specific relationships between people and their environments, it is possible to develop an approximation of ‘how people relate to *everything* that bears upon their lives’ (Miller, 2018: 6; emphasis added) including, but never just exclusively, smartphones. Thus, it becomes possible to move beyond insights from more ‘universalising disciplines’ (Miller, 2018: 6) about smartphone lives and generate insights about how the diverse smartphone shapes, and is shaped by, its embedding in diverse settings.

In our own research this is exemplified in how smartphones have been transforming moral mobilities in the Lau Lagoon. As we walked through villages in the rural Lau Lagoon, Solomon Islands, we quickly learnt that passers-by are regularly asked not ‘how are you doing?’ but ‘where are you going?’ or ‘where are you coming from?’ This line of questioning allows for understanding the moral frameworks that surround individuals’ mobility in rural environments. Passers-by are expected to disclose the origins, and destinations, of their movements as a way to assert the morality of their motivations for movement; and if they do not respond to such requests, individuals’ movements are treated with suspicion. A failure to respond to questions about the place in question suggests, in the Lau Lagoon, for instance, that the passer-by has something to hide and this ‘something’ could be as substantive as an extramarital affair, one of the most common sources of conflict in the region.

Smartphones variously disrupt this framework for moral mobilities. During our 2014–2015 fieldwork, we noticed how some villagers moved through the village immersed in their smartphones. These smartphone users were not busy with a call, but were absorbed in a ‘privatized auditory bubble’ (Bull, 2005: 344). Individuals walked through rural spaces listening to personalized playlists, often using loudspeakers on a somewhat quiet setting (headphones were not widespread during our participant observation). While listening to music, these smartphone users regularly ignored calls from others asking ‘where they are going?’ or ‘where are you coming from?.’ These smartphone mobilities, thus, became a ‘practice of transgressive… walking’ (Coates, 2017: 28). They facilitated a heightened sense of uncertainty, or dissociation, in the village and lead to calls for banning listening to music in public settings while on the move. Villagers were worried that practice may lead to (potentially unsubstantiated) accusations of moral impropriety, even resulting in (potentially unnecessary) conflict, thus, disrupting village life.

Without being there ourselves, we could not have hardly uncovered how substantive a threat ‘auditory bubbles’ are to social cohesion in the Lau Lagoon, and how they are embedded in broader debates about the morality of individuals’ mobilities; and without this careful attention to shifting moral mobilities we may also not have noticed how they help understanding broader dynamics such as why, when and how the Lau use or reject so-called ‘development infrastructures’ such as roads (cf Hobbis, 2019b). We needed to observe, and participate in what it meant to move through villages and between them, as much as possible as the Lau do. By so doing we were able to gain insights into related verbal and nonverbal knowledge ‘[absorbing] also through the skin, and [learning] through all the senses’ (Okely, 2007: 75), developing our own embodied experience of our topics of inquiry.

Participant observation, as envisioned by this approach, accordingly encompasses all aspects of life and not just focusing on those aspects that most explicitly link to smartphones. For example, in our case it also meant learning not just how to move with and like the Lau but also the body techniques for how to garden, how to cook local foods, and even how to weave mats. It meant learning how to celebrate a marriage or birth, and how to respectfully mourn; and it meant more broadly, to learn the nuances of everyday life including mobilities but also, among others, how to share goods in a complex system of giving and counter-giving. Doing so allowed for uncovering the broader embedding of particular body techniques and, thus, eventually for understanding why and how the Lau was so distraught by shifting mobility patterns as a result of smartphone-enabled ‘auditory bubbles,’ among many other moral concerns that we uncovered (Hobbis, 2017, 2020).

Finally, participant observation should ideally take place over an extended period of time, despite or rather in opposition to tendencies of ever accelerating research speeds in neoliberal environments (cf Rasch et al., 2022). By slowing down with data collection (Grandia, 2015), the smartphone researcher can make space for more meaningful engagements with research participants. They can develop a more carefully co-produced understanding of the sociotechnical context in which the research takes place, all while establishing rapport with participants that might allow insights into the more intimate dimensions of smartphone use (e.g., the extramarital affairs that Lau villagers worried about). As such the smartphone researcher may, especially if the work takes place in remote, marginalized contexts, aim towards addressing the extractivist, exploitative, tendencies of ‘hit-and-run’ relationships (Wax, 1997: 55 cited in Grandia, 2015: 312) found in more hurried research. Simultaneously, it allows for gaining the in-depth insights needed for the kind of analysis that allows for shifting beyond the ‘average.’ It allows for, in media research in particular, uncovering the ‘small but necessary details that render the materiality of media (and hence its particular affordances and constraints) not only heterogeneous but fully cultural, social, and… political’ (Coleman, 2010: 491).

### Smartphone-centric interviews

Participant observation offers a more holistic, longitudinal understanding of everyday life, and an embodied understanding of smartphones in this life. This understanding constitutes the basis for interviews as second key method of our sociotechnical approach. These interviews—in our case, initiated after eight months in the field in the tradition of ‘classic’ or ‘slow’ ethnographic research—aim to understand smartphones in all their temporal and spatial entanglements. They extend understandings of the smartphone researcher into, for example, auditory bubbles, and, more broadly, into areas not (easily) observable in everyday interactions. In addition, they are the cornerstone of the media-centric dimensions of this sociotechnical approach. By being materially grounded in (questions about) the smartphone these interviews acknowledge the supercompositionality of smartphones, the ability of smartphones to bridge social networks and cultural meanings on a sociocultural *and* technological level. The interviews themselves are subdivided into three steps, each accompanying broader, related insights from participant observation, and each addressing broader questions surrounding material cultures, body techniques and sociotechnical systems.

Overall, these interviews open up an extraordinary degree of access to the private lives of participants, thus, necessitating equally extraordinary ethical safeguards. Hence, we did not only discuss our general research goals as participant observers and ahead of the interview but also the particularities of each step of the interview in advance with each participant, giving participants the opportunity to carefully select what information they wanted to share with us and also to withdraw their consent at any time. For example, we asked participants to preselect which functions and files they did, or did not, want to share. In our case this was no problem and even a very familiar process for our participants. While phones are generally individually owned they are also regularly shared with others who, for instance, do not have the power to operate their phone or who do not own a phone at that particular moment in time. Since phones are, therefore, not primarily ‘private’ our participants have developed various techniques for ensuring that some digital files and usages are not easily identifiable by others operating the same phone, from storing some phone numbers in paper-based notebooks only to maintaining a second, ‘for their eyes only’ microSD card.

#### Step 1: object itineraries

An elaboration on object biographies, step 1 of the smartphone-centric interview uncovers ‘object itineraries’ (Bauer, 2019; Joyce and Gillespie, 2015). Object itineraries trace ‘the routes through which [smartphones] circulate, and the means by which they are moved’ (Joyce and Gillespie, 2015: 1). Accordingly, step 1 asks participants to provide a history of their lives with their current and previous smart- and more broadly mobile phones. Questions include how many phones they had previously owned, their makes and models as well as what they thought about these makes and models, their likes and dislikes. Simultaneously, questions ask where and when participants had acquired and used individual phones, what threats they faced to their survival and where, when and how these phones had eventually died. Through these questions it is possible to gain an understanding of the user's broader economic and social life history as it relates to smartphone and broader technological interests such as shifting desires for electricity infrastructures (cf Hobbis, 2021).

Simultaneously, ‘object itineraries’ recognize that even though materials such as smartphones are intrinsically mobile, their mobilities are configured and enabled through ‘spatial, infrastructural and institutional moorings’ (Hannam et al., 2006: 3). These moorings require objects to come to a temporary stop in at least one core aspect of their mobility—e.g., at the places that smartphones are sold in Solomon Islands or at the homes of their eventual owners—and are analytically especially interesting because they create particularly visible intersections with other itineraries, especially those of everyday life. Even when objects have come to a halt, they are ‘still in motion relative to the seasons, the air, and other things (animals, humans, objects, etc.) whose own itineraries it intersects’ (Bauer, 2019: 343) and they are uniquely rather than deterministically so. For example, a particular smartphone may cease to function because it is submerged by saltwater during a canoe ride to a market, or because it breaks, falling out of a pocket while climbing a coconut tree to get better reception (cf Hobbis and Hobbis, 2021).

Object itineraries allow for recognizing such variance. The same type of object may follow different trajectories and intersect differently with other itineraries (Bauer, 2019; Joyce and Gillespie, 2015). By not presupposing a particular trajectory, object itineraries necessarily uncover multivalent presents. By so doing, they are useful to move beyond generalised understandings of smartphones and how they are part of everyday life in particular contexts and places. They allow for analysing how smartphones are *actually* used, in our case, in Solomon Islands, while recognizing that this use is intrinsically entwined with ‘the stories of what happens to [smartphone materials] as they flow, mix and mutate’ (Ingold, 2007: 14).

Step 1, thus, provides central insights into the broader sociotechnical contexts in which mobile devices are acquired, maintained, used and eventually discarded. Among the Lau, interviews focused on object itineraries allowed us to deepen our understanding of the vulnerability of smartphones in Solomon Islands. They revealed how easily smartphones die in this tropical environment, just because of the humidity, the sand or exposure to saltwater (cf Hobbis and Hobbis, 2021). At the same time, they revealed localized perspectives on this vulnerability that are deeply embedded in broader sociotechnical systems. These sociotechnical systems accept and expect materials as transient in everyday life, and in their transience as ‘another opportunity for relationship-building through exchanges’ (Hobbis, 2021: 162). For instance, a new phone as a gift from an urban relative helps strengthen social networks, similar to, and in some cases perhaps even more so, than regular phone calls between the same relatives as giving and receiving constitutes.

By uncovering moments of exchangeability, expressed in when, how and why phones are acquired, maintained and discarded, step 1 furthers understandings of the social relevance of smartphones as embedded in particular sociotechnical systems. Simultaneously, they provide a general context for phones and their operators, situating them in broader debates about when, why, and how people choose to invest, often limited financial, but also social, resources, into (particular) smartphones, at particular moments in time.

#### Step 2: body techniques

Step 2 of the smartphone-centric interview narrows in on the centrality of ‘body techniques’ or ‘the ways in which from society to society [people] know how to use their bodies’ (Mauss, 2006: 70). This means shifting away from a focus on the macro-movements of phones and their users, as they may be uncovered in object itineraries, towards micro-movements. Micro-movements include, among many others, actions such as swiping screens with forefingers to refresh social media or typing with phones in what Glotz and colleagues call ‘thumb culture’ (2005) and Nicholas Nova et al. (2012) refer to as ‘curious rituals.’ Examples range from ‘clicker casting,’ when users shift their wrist position when trying to broadcast the transmission from television remote controllers to the receiving sensor, to ‘the periscope,’ the move people make when taking pictures or videos with phones or tablets held up above their heads (Nova et al., 2012).

Many of these body techniques can be observed and experienced during participant observation. Interviews serve the purpose of developing a more concise understanding of these body techniques, to confirm and/or challenge the researcher's interpretation of observations. Simultaneously, they help the researcher to gain additional insights e.g., into even more microscopic or purposefully hidden body techniques. For instance, Geoffrey kept coming across the same artifact in his survey of Lau MicroSD cards, particularly among men: an untitled folder which contained another untitled folder which contained another untitled folder and so on. Dozens and dozens at a time. At the bottom, he regularly found a collection of sexually suggestive foreign-made music videos. Hiding these files is crucial. If the files were publicly accessible they are feared to lead to a broader moral deterioration that secrecy seeks to prevent.

By creating a seemingly never-ending chain of folders, the goal was to simply wear out possible users. Unintended users might click on the folder a few times but eventually, they would give up. The goal was to manage, in as far as possible, the socially destructive potential of digital technologies and in this case digital media to social life and the relationships of a given microSD cards’ owner. After all, MicroSDs are often enough at least partially shared—a child or spouse or other family member may borrow the phone that contains it or even the MicroSD itself. In this case, digital files are managed to allow for individual enjoyment and for the relationships they foster—sharing such a file with another man can help one's social standing—while not disrupting the familial relationships that such files are thought to destroy only too easily (cf Hobbis and Hobbis, 2022b). At the same time, the technique itself is only truly hidden to those who do not know about it. Once revealed to Geoffrey as part of these interviews, he learnt to identify rapid, repeat, nearly rhythmic tapping of the smartphone screen as a user likely accessing such hidden files, becoming, with this knowledge, further embedded in men's embodied, everyday lives.

Other body techniques became apparent during interviews as well, not because they are necessarily purposefully hidden, but because they are similarly small in scale. For instance, there are body techniques ‘involved in the making, use, and repair of things’ (Kuipers et al., 2018: 236) that ‘recede into the background until they break or a tension emerges requiring attention to their material features’ (Kuipers et al., 2018: 238). Exemplary in our context are gestures linked to the insertion of microSD and SIM cards in a context where they perpetually meet other, largely corrosive materials, from sand to saltwater. These techniques such as putting a piece of paper between microSD cards and the metal buckles that are meant to keep it in place, often serve the purpose of keeping phones alive longer. They highlighted, in our case, Lau capacities to maintain the functionality of their smartphones, despite no formal training in doing so and reflecting a broader technological ingenuity among the Lau who are not only skilled fisherfolk but who have a longstanding history of building and maintaining their own artificial islands (cf Ivens, 1930).

Step 2 allows for uncovering the immediate entanglements of smartphones and their users’ bodies, without necessarily involving any form of e.g., self-tracking software. Some of the techniques linked to everyday encounters with hardware, including repair, will be in articulation to the simple operation of the technology. Others, however, are not. They offer, such as the hidden files, further insights into the contextually-specific social embedding of smartphones. This is what anthropologists of technology take to be speaking towards geographically particular social representations (Kuipers et al., 2018; Lemonnier, 2012). It sets up the possibilities for novel comparisons that highlight sociocultural particulars in spite of the seeming universalizing effects of globally ubiquitous technologies such as smartphones.

#### Step 3: smartphone materialities

Step 3 of the smartphone-centric interview ‘gets into’ each interviewee's smartphone materiality as comprehensively as possible. Here, our approach builds on Ito et al.'s (2009) ‘Portable Kit Study’ and its adaptation by Taylor and Horst (2013, 2014). The primary innovation is that rather than interviewing participants about all the objects they carry on them in ‘their bags, pockets, and wallets’ (Horst and Taylor, 2014: 160), our sociotechnical approach treat smartphones themselves as a type of digital pocket that contain ‘a broad range of material practices’ (Taylor and Horst, 2013: 92). After all, smartphones are a ‘metamedium’ (Humphreys et al., 2018) with a multitude of diverse functions, from telecommunication to entertainment to time measurement.

Centrally, for this sociotechnical approach to interviewing all functions and data are to be considered individually and in relation to each other to uncover ‘the repertoires of practices and meanings that emerged from their collective use’ (Horst and Taylor, 2014: 160). Questions include when, why and how installed smartphone apps or functions are being used. The interviewer walks with interviewees through all the functions and data that their devices contain, irrespective of any assumed popularity of one function over another. In the vein of walkthrough methods more broadly, ‘the core of this method involves the step-by-step observation and documentation of an app's screens, features and flows of activity—slowing down the mundane actions and interactions that form part of normal app use in order to make them salient and therefore available for critical analysis’ (Light et al., 2018: 881). Notably, if apps were pre-installed but not used, the interviewer also asks about individuals’ knowledge of, and thoughts about, these apps and their (presumed) users.

Doing so furthers the efforts of this approach to approximate a more holistic understanding of the smartphone in everyday life, and everyday life as it now includes the smartphone. By giving equal value to each app/function, part 3 of the interview may reaffirm presumptions about popular dimensions of smartphones. Yet, it may also uncover more unexpected dimensions as they are again embedded in a particular sociotechnical setting. For instance, when Stephanie was in Solomon Islands in 2018, most people she talked to had installed the seemingly mundane SHAREit app on their smartphones. SHAREit allows for offline transfer of digital files similar to Bluetooth, but considerably faster—according to the developer, at 200 times the speed. As we have described elsewhere (Hobbis and Hobbis, 2022b), Solomon Islanders actively share multimedia files across smartphones as part of broader exchange relationships, strengthening social networks between rural and urban environments (cf Crowdy and Horst, 2022). The problem with Bluetooth, preinstalled on most phones we encountered, is that sharing larger files, such as a movie, can take hours and possibly consume more battery power than available. This makes it difficult, or even impossible, to transfer some desired files. The SHAREit app removes this obstacle. It, thus, advances the circulation of digital gifts for social reproduction and had, at least in 2018, become one of the most highly valued apps on Solomon Islanders’ smartphones—for its specific, technological moral embedding. In other words, uncovering expected and unexpected usages of any app installed on, or function available on, a given smartphone, the researcher takes seriously the moral context in which smartphones are adopted and adapted, and which they strengthen or potentially undermine.

Centrally, step 3 not only walks systematically through apps/functions but also through any files stored on a given phone or microSD cards as additional material traces of users’ activities. By so doing, smartphone researchers are able to shift beyond a preoccupation with often pay-per-use communicative functions. In Solomon Islands this was especially significant. Most of our respondents were unable to regularly afford call/text credit or data and therefore they preferred those aspects of smartphones that could be used ‘for free’ and that ‘merely’ required enough battery power to run them. These included the aforementioned basic functions as well as a diverse digital entertainment files.

By carefully going through each file in a person's microSD card, we were able to develop a better understanding of the relationship between foreign and locally produced files, the particular values ascribed to them and how these values link to smartphone materialities. We realised, among others, that the majority of files on villagers’ smartphones were foreign ones, with especially family pictures and videos being comparatively rare and regularly deleted. At first sight this seemed to contradict research on photography elsewhere in Solomon Islands (Wright, 2013), which has argued to materialise especially family attachment across space and time and even beyond death. However, by accounting for villagers’ understandings of the materialities of digital files, we learnt that villagers expect not only their phones but also their microSD cards and digital files to decay rather sooner than later, for instance, because of the many computer viruses that accompany foreign files as they are shared between microSDs (Hobbis and Hobbis, 2021). As a result, villagers continue to print and laminate their most treasured family photographs with the process of lamination being considered to be considerably more durable than the digital storage facilities available to them (Hobbis and Hobbis, 2021; Wright, 2013). In other words, by materially grounding our analysis of digital multimedia files, we were, among others, able to challenge the myth of digital durability that surrounds data as ‘divorced from the everyday dirt and matter of daily life’ (Bollmer, 2015: 66) and that is closely linked to media-centric studies of media as focused on its virtual rather than material dimensions (Morley, 2009).

Step 3 also acknowledges the problem with self-reported use of particular apps/functions (cf Boase and Ling, 2013). In as far as it is possible without passive data collection, grounds interviews in discussions of not just apps and functions but also associated logs, e.g., as they accompany ‘call’ and ‘text histories.’ In our case, we started, for call and text histories, with the first entry and work our way systematically from beginning to end. We would do the same with the phone book, a record of people including their contact information for easy access and identification when making and receiving calls/texts. For each entry we asked: Who is this person? How long have you known them? When did you meet them? Where are they based? Why did you call/text them in this instance? Why did they call/text you in this instance? How often do you call/text them? How often do they call/text you? Why do you usually call/text them, or they call/text you? This task that was substantive but still doable. Most of our interviewees used their phones for calls/text more often than once a week, if at all, and most phones had been acquired within the year preceding the interview. In other contexts, the scope of the included logs may be temporally restricted, e.g., by focusing on the previous month or two, as is common even with passive data collection via logs.

By materially grounding sociotechnical interviews in these logs, it is possible to create more reliable data points on phone use, with full consent of participants throughout. For example, whenever an interviewee did not want to disclose why they had called a particular number, we would jump ahead to the next, excluding this particular instance from our data collection. On a different level, we were able to more reliably uncover instances of shared phone use. Phone sharing is mostly hidden by automated, passive data collection but widespread in everyday life. David Lipset has, for example, documented it in some areas of neighbouring Papua New Guinea (2018) and Miller et al. (2021) note that phone sharing is a common practice in Kampala, Uganda. Indeed, sometimes our participants did not know who was called or even who a person in their contact list was. However, they would be able to provide insights into who else had used the particular headset, and at least approximate, based on available logs or other material traces such as multimedia files unknown to the interviewee, how often and for what reasons they had done so.

The emerging materially grounded interview data then allows for comparability across diverse cultural contexts. For example, our findings echo Daniella Kraemer's observations in Vanuatu that urbanites, in our case including older generations, use mobile telephony to ‘[extend] the breadth of social relationships’ (2017: 43), be it through cold calling or by adding numbers of school friends or market acquaintances (Hobbis, 2020). However, we also found that, at least among the Lau, this ‘extension’ does not occur during stays in rural areas highlighting the particular rural dimensions of smartphone use (Hobbis, 2020). Some of our interviewees still had the phone numbers of urban friends saved in their phone books, often, as a ‘repository’ in anticipation of a return to town. When in village environments, they did not call these numbers and they had also not added any new ones as indicators of new social relationships beyond kin groups. Instead, we found that rural residents used mobile telephony, first and foremost, for maintaining the breadth of existing kin rather than broader social relations.

Finally, step 3 recognizes the materiality of the smartphone at its most basic level. It means literally, in as far as reasonable, disassembling the handset hardware together and talk about the constituent pieces and the symbols on them (batteries, SIM cards, MicroSD cards, screens, touch pads, etc.). This brings further attention to more mundane, yet for the everyday usability of mobile phones indispensable materialities, allowing, in particular, for a better understanding of how people conceptualise the mechanics of smartphones. Simultaneously and building on findings on maintenance and decay from parts 1 and 2 of the interview, this step further situates smartphones in broader histories of technological maintenance and possible decay, itself a new ‘agenda for interpretation, reinterpretation, and new research for historians [and ethnographers] of technology’ (Russell and Vinsel, 2018: 3; cf Kuipers et al., 2018).

## Concluding reflections

Combined, participant observation, with a particular interest in body techniques, and the smartphone-centric interview offer an opportunity to crystalize the strengths of both types of both media- and non-media centric approaches to understanding the smartphone in everyday life *and* to understanding everyday life in the smartphone age. They are exemplary for a mobile method that emphasises the value of ‘the collection of data *in situ,* that is, the collection of data in particular places or moments of meaning’ (Boase and Humphreys, 2018: 155), thus, offering insights into the diversity of smartphones around the world.

At the same time, by avoiding pre-assigning degrees of significance to the smartphone and its individual functions at any given place and time, our sociotechnical approach allows for the kind of flexibility needed when the technology and its uses can change rapidly. By going through the materiality of the smartphone step-by-step the smartphone as it is being used at a particular place, and time, is revealed; whatever the particular technological characteristics a smartphone may have at that place and time. In other words, our approach is intensely adaptable focused on the materiality of what is, rather than what is assumed to be, often, from afar.

Flexibly grounded in this materiality, our approach then also recognizes that smartphone worlds, in particular places and in comparison, are always necessarily in *medias res, ‘*contingent, dynamic, multiple and indeterminate’ (Orlikowski, 2007: 1145). Our approach requires the smartphone researcher to engage in a constantly evolving process of refinement, as is common for slow, longitudinal fieldwork (cf Varisco, 2019). A first field experience yields a different level of result than a second, or third; and so too is the deployment of our approach to smartphone research. What the practitioner of this approach must know is that they are responsible, based on their on-the-ground judgement, for ascertaining the threshold of data saturation, at particular moments in time, necessary for illuminating the insights generated from smartphone research.

Finally, our approach highlights and draws upon smartphones as excellent artifacts for social inquiry, in contexts of potentially rapid changes. Through their very materiality, smartphones computationally extend, more broadly, our ability ‘to describe the lives of people other than ourselves, with an accuracy and sensitivity honed by detailed observation and prolonged first-hand experience’ (Ingold, 2008: 69). Simultaneously, this approach provides a materially-grounded opportunity for ‘generous, comparative but nevertheless critical understanding of human being and knowing in the one world we all inhabit’ (Ingold, 2008: 69). It contributes a technique for the comparative use of smartphones to narratively map the material traces of social interactions that human users leave on smartphones without the intrusiveness of passive data collection methods that may be ethically questionable in many contexts with long histories of (data) extraction and colonization. By locating and analyzing these traces, we contend that this approach is then also a step towards breaking ontological barriers (Hirsch and Rollason, 2019). It provides not only an ability to understand the role that smartphones play in everyday life in particular contexts but also crucial missing empirical evidence for efforts aimed at retheorizing ‘shared worlds’ (Pina-Cabra, 2014a, 2014b)—smartphone worlds—‘to take account of the evident and effective connections between peoples—even those who seem very different from one another’ (Hirsch and Rollason, 2019: 10).

## Notes

1. To a lesser but still notable extent, smartphones are increasingly also considered for disseminating research results, e.g. as access points for digital archives and as means for digital repatriation (cf Bell et al., 2013).

2. Not all smartphones are the same, and they are designed for different markets and considering different needs far beyond the Global North (Lu, 2021).

3. See Brunn and Wahlberg (2022) for a discussion of the dominant, partially distinct, French, US American and British anthropologies of technology.
